# Insights into CoFe_2_O_4_/Peracetic Acid Catalytic Oxidation Process for Iopamidol Degradation: Performance, Mechanisms, and I-DBP Formation Control

**DOI:** 10.3390/nano15120897

**Published:** 2025-06-10

**Authors:** Haiwei Wu, Jiaming Zhang, Fangbo Zhao, Wei Fan, Song Yang, Jun Ma

**Affiliations:** 1 College of Material Science and Chemical Engineering, Harbin Engineering University, Harbin 150001, China; 2 School of Environment, Northeast Normal University, 2555 Jingyue Street, Changchun 130117, China; 3 Resources and Environmental Innovation Institute, Shandong Jianzhu University, Jinan 250101, China; 4 School of Environment, Harbin Institute of Technology, Harbin 150001, China

**Keywords:** peracetic acid, CoFe_2_O_4_, iopamidol, metastable intermediate, monoiodoacetic acid formation potential

## Abstract

In chlorination disinfection treatment, residual iodinated X-ray contrast media (ICMs) are the precursors to iodinated disinfection by-products (I-DBPs). This study employed CoFe_2_O_4_ nanoparticle catalytic peracetic acid oxidation (CoFe_2_O_4_/PAA) to remove iopamidol (IPM) and control I-DBP formation. The experimental results demonstrated that over 90% of the IPM degradation was achieved in 40 min. The metastable intermediate (≡Co(II)-OO(O)CCH_3_), rather than the alkoxyl radicals, was identified as the dominant oxidation species (ROS). The electron transfer pathways between the metastable intermediate and IPM were oxygen-atom transfer and single-electron transfer. The monoiodoacetic acid formation potential (MIAAFP) was investigated. In ultraviolet-activated ClO^−^ (UV/chlorine), a portion of I^−^ generated through IPM dehalogenation underwent conversion to reactive iodine species (RIS), consequently elevating the MIAAFP. In CoFe_2_O_4_/PAA, the MIAAFP was less than 43% of that in UV/chlorine, which can be attributed to the complete conversion of I^−^ into iodate IO_3_^−^ without generating RIS. CoFe_2_O_4_/PAA is thus a promising treatment for removing ICMs and controlling I-DBP formation due to the efficient degradation of ICMs while avoiding the generation of RIS.

## 1. Introduction

Iodinated X-ray contrast media (ICMs) are widely employed in human diagnostic tests, particularly enhanced computed tomography and magnetic resonance imaging [1]. The global ICMs market was USD 5.23 billion in 2019 and is expected to reach USD 6.9 billion by 2027 [2]. As ICMs are minimally metabolized by the human body, most are released into hospital wastewater and the ecological environment [3]. Furthermore, owing to their high water solubility and low biodegradability, ICMs are difficult for conventional wastewater treatment to remove. Consequently, ICMs are frequently detected in the outlets of municipal wastewater plants, groundwater, and even drinking water, with concentrations reaching 10^3^ μg/L [4,5,6]. Moreover, the residual ICMs are regarded as the precursor to iodinated disinfection by-products (I-DBPs), which are partially converted into I-DBPs in the chlorination disinfection process [7]. The I-DBPs are more genotoxic and cytotoxic than chlorinated and brominated disinfection by-products (Cl-DBPs and Br-DBPs) [8].

Among the novel ICM removal methods, advanced oxidation processes (AOPs) have demonstrated promising application prospects. It has been reported that an ultraviolet-activated chlorine (UV/chlorine) system can effectively remove iodohexanol from water supply systems [9]. Most AOPs, such as Fenton-like treatments and visible-light-enhanced catalytic oxidation, have sufficient oxidation capacity for ICM removal [10,11]. However, AOPs also have limitations. The competitive consumption of hydroxyl radicals (HO•) between dissolved organic matter (DOM) and ICMs manifests as a radical scavenging effect, with DOM preferentially sequestering reactive oxygen species, thereby depleting the radical availability and leading to a reduction in the ICM elimination efficiency. In addition, reactive iodine species (RIS) generated from the dehalogenation process, including I_3_^−^, I_2_, I•, I_2_•^−^ and HIO, accumulate and react with DOM, increasing the risk of I-DBP formation [12,13]. Therefore, developing novel AOPs that can remove ICMs while avoiding RIS generation is of considerable importance.

Peracetic acid (CH_3_CO_3_H, PAA) has been employed as an effective oxidant for disinfection and oxidation owing to its high oxidation potential of up to 1.96 V [14]. The O-O band of PAA breaks easily to generate HO• and alkoxyl radicals (R-O•, mainly CH_3_CO_2_• and CH_3_CO_3_•); thus, the AOPs based on PAA activation have been applied in water treatments [15,16]. Cobalt (Co) is known as an efficient transition metal activator of PAA, outperforming other transition metals, such as manganese, iron, and copper [15]. Therefore, Co/PAA is a promising treatment for removing organic contaminants. Ferrates (MFe_2_O_4_, M = Cu, Co, Ni, etc.) are spinel-structured compounds that exhibit excellent mechanical and chemical stability. The ferromagnetism makes it feasible to separate them from the reaction system [17]. Owing to these advantages, ferrates have been widely applied in catalytic oxidation processes. In addition, it has been reported that CoFe_2_O_4_ can be reused in practical applications, which considerably reduces the economic costs in industries [18]. Therefore, the CoFe_2_O_4_/PAA oxidation system is expected to demonstrate value in industrial applications.

In the PAA disinfection process, HBrO and HClO will not accumulate, and the generation risk of Cl-DBPs and Br-DBPs is significantly lower than that of traditional chlorination disinfection [19]. However, significant knowledge gaps persist regarding the PAA-mediated degradation mechanisms of ICMs and subsequent I-DBP formation. Herein, iopamidol (IPM), a prevalent ICM species ubiquitously detected in aquatic environments [20], is selected as the target contaminant in this study. CoFe_2_O_4_/PAA is employed to achieve the following objectives: (i) assess the IPM removal efficiency in CoFe_2_O_4_/PAA, (ii) investigate the oxidation mechanisms of CoFe_2_O_4_/PAA by identifying the dominant oxidant and analyzing the electron transfer pathways, and (iii) evaluate the formation of monoiodoacetic acid (MIAA).

## 2. Experimental Methods

### 2.1. Chemical Reagents and Materials

The chemical reagents in this study were of analytical purity. The details of all the chemical reagents are described in Table S1. The CoFe_2_O_4_ nanoparticles were purchased from Aladdin Biological Technology Co., Ltd. (Art. No. C683798, Shanghai, China). The average particle size of the CoFe_2_O_4_ was 342 nm (Figure S1). Surface water samples were taken from Songhua River in Harbin, and the water quality parameters were provided in Table S2. The surface water samples were filtered through 0.45 µm membranes before the oxidation experiments. The concentration of IPM was set at 2 µM.

### 2.2. Experimental Procedures

PAA was prepared from the reaction between CH_3_COOH and H_2_O_2_, with concentrated sulfuric acid (H_2_SO_4_, ≥98%) used as the catalyst. First, 1 mL H_2_SO_4_ was added slowly into a 250 mL beaker with 45 mL CH_3_COOH under magnetic stirring at room temperature. Then, 40 mL of 30% H_2_O_2_ was added dropwise to make the reaction smooth. The stock solution was set aside before use for at least 48 h to balance the reaction, and it was stored at 4 °C [21].

The humic acid (HA) stock solution was prepared by vacuum filtration (0.45 μm fiberglass membrane) and stored at 4 °C; the concentration was determined by its total organic carbon (TOC). The RIS was captured by the phenol, and the experimental procedure is described in Text S1.

The CoFe_2_O_4_/PAA system oxidation experiments were conducted in 250 mL beakers at 25 ± 2 °C. The appropriate amount of PAA and IPM stock solution was successively added into the beakers under mechanical stirring. The pH of the experiments was adjusted by adding 0.1 M of H_2_SO_4_ and NaOH solution. The addition of CoFe_2_O_4_ initiated the oxidation experiments. The samples were withdrawn at preset time intervals and put into 10 mL sample bottles to which excess sodium thiosulfate had already been added to quench the oxidation reaction. The sample solution was filtered into a 2 mL HPLC sample bottle (with a 0.45 μm fiberglass membrane).

The oxidation experiments in the UV/PAA and UV/chlorine systems were performed in a 20 mL quartz dish (diameter of 5 cm) under stirring at 25 ± 2 °C. The low-pressure mercury lamp (Heraeus, Hanau, Germany, 10 W GPH 212T5 L/4) emitting light at 254 nm was vertically 10 cm above the liquid surface in a shuttered box. The average fluency rate was 1.54 mW/cm^2^, as measured by a UVC radiometer (Optical Instrument Factory of Beijing Normal University). The lamp was turned on 10 min before the reaction started to ensure the light was stable. The samples were withdrawn at preset time intervals and put into HPLC sample bottles with excess sodium thiosulfate to quench the oxidation reaction. Each experiment was conducted in triplicate, and the mean values with the standard deviations were presented.

The disinfection process was performed with sodium hypochlorite. The oxidation samples of CoFe_2_O_4_/PAA were filtered to remove the catalyst (by a 0.45 μm fiberglass membrane). The samples of UV/chlorine did not require any pretreatment. After extracting 10 mL oxidation samples into 40 mL amber glass bottles with polytetrafluoroethylene-lined septum screw-caps, 15 mg/L of sodium hypochlorite was added. The samples were kept in the dark for 48 h. Each experiment was conducted in triplicate, and the mean values were reported, and the error bars were within the 95% confidence interval.

### 2.3. Analysis Methods and Instruments

The element contents and crystal morphological structure of the catalyst CoFe_2_O_4_ were analyzed by X-ray photoelectron spectroscopy (XPS, XSAM 800). The C 1s peak corrected the binding energy of each element. Then, the peaks were separated by a Gaussian fit. The particle size of CoFe_2_O_4_ was measured using a Malvern Nanosizer (Zetasizer Nano ZS 90, Malvern, UK). The powder X-ray diffraction (XRD) data were obtained on a RigakuD/MAXRC X-ray diffractometer with a Cu Ka radiation source (45.0 kV, 50.0 mA). The concentration of PAA and H_2_O_2_ in the PAA stock solution (high concentration) was detected via titration, and the PAA concentration in the reaction solution (low concentration) was determined via a modified spectrophotometric determination method [22], and the ratio of PAA to H_2_O_2_ in the PAA stock solution was 1:1.38.

The residual of organic compounds, the concentration of I^−^ and IO_3_^−^, was measured by high-performance liquid chromatography (HPLC, Agilent 1260 Infinity II) with a C18 column (4.6 × 250 mm, 5 μm, Waters, PN.186003117). The detailed mobile phase and wavelength for detection are shown in Table S3. The total organic carbon (TOC) was measured with a Multi 3100 N/C TOC analyzer. The UV-Vis absorbance and absorption spectra were measured via UV/Vis spectrophotometry (Hatch-DR5000, Mississauga, ON, Canada). The reaction pH was measured by a pH meter (E-201-C). The electron spin resonance spectrometer (ESR, A200, Bruker, Billerica, MA, USA) was used to detect radicals and ^1^O_2_, using 5,5-dimethyl-1-pyrrolidine N-oxide (DMPO) and 2,2,6,6-tetramethyl-4-piperidinol (TEMP) as the trapping agents. The concentrations of Co and Fe were determined by an ICP–mass spectrometry (NexION^TM^ 300Q, Pyrmont, NSW, Canada). The oxidation species were analyzed by liquid chromatography–mass spectrometry (AB SCIEX, TripleTOF 5600, Marlborough, MA, USA), and the detection method is shown in Text S2. Electrochemical measurements were conducted in a three-electrode electrochemical cell equipped with an electrochemical workstation (Metrohm, PGSTAT302N, Herisau, Switzerland). The nickel foam electrode (NFE) coated with CoFe_2_O_4_ was prepared as the working electrode. The reference and counter electrodes were the Hg/Hg_2_Cl_2_ and platinum electrodes, respectively. The procedure of the gas chromatograph–mass spectrometer (GC-MS) for the MIAA detection is provided in Text S3. The results of the N_2_ adsorption experiments were analyzed by a specific surface area and porosity analyzer (TriStar II 3020, Culver City, CA, USA).

## 3. Results and Discussion

### 3.1. Characterization of CoFe_2_O_4_ Nanoparticles

Figure S2a shows that the characteristic peaks at 30.0°, 35.5°, 43.3°, 57.1°, and 62.6° were well matched with the standard XRD pattern of CoFe_2_O_4_ (JCPDS 22-1086) [23]. CoFe_2_O_4_ showed typical type IV isotherms with a H3-type mesoporous hysteresis loop (Figure S2b), which is characteristic of mesoporous materials. The specific surface area, total pore volume, and average pore diameter of the CoFe_2_O_4_ particles were 21.153 m^2^ g^−1^, 0.116 cm^3^ g^−1^, and 17.076 nm, respectively.

### 3.2. IPM Degradation Efficiency in CoFe_2_O_4_/PAA

**Figure 1:**
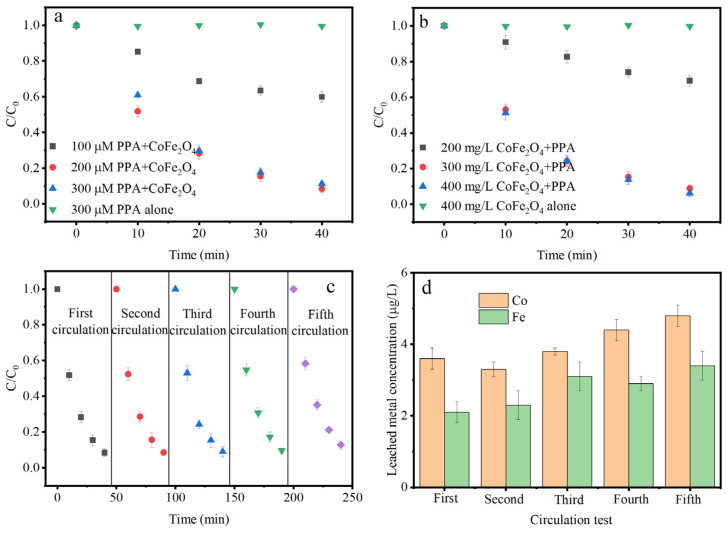
Effect of PAA dosage (**a**), effect of CoFe_2_O_4_ dosage (**b**), multiple oxidation efficiency (**c**), and metal leaching in CoFe_2_O_4_/PAA (**d**). Conditions: (**a**) [IPM] = 2 μM, [PAA] = 100–300 μM, CoFe_2_O_4_ dosage = 300 mg/L, and pH = 7.0; (**b**) [IPM] = 2 μM, [PAA] = 200 μM, CoFe_2_O_4_ dosage = 100–400 mg/L, and pH = 7.0; and (**c**,**d**) [PAA] = 200 μM, dosage of CoFe_2_O_4_ = 300 mg/L, [IPM] = 2 μM and pH = 7.0.

Figure 1a,b illustrate that the IPM removal rate was <5% when using PAA and CoFe_2_O_4_ alone. Therefore, the oxidation capacity of PAA and the adsorption of CoFe_2_O_4_ to IPM were negligible. When increasing the PAA concentration from 100 to 200 μM, the IPM removal rate increased from 40.1% to 91.1%. However, at a PAA concentration of 300 μM, the change in the IPM removal can be ignored (Figure 1a). At a CoFe_2_O_4_ dosage of 400 mg/L, the IPM removal rate only increased by 1.2% compared to that at a CoFe_2_O_4_ dosage of 300 mg/L. Therefore, the optimal process parameters at a neutral pH were determined to be 200 μM PAA and 300 mg/L CoFe_2_O_4_. Considering that the ratio of PAA to H_2_O_2_ was 1:1.38, 276 μM H_2_O_2_ was obtained in a 200 μM PAA solution. Figure S3 demonstrates that the IPM removal rate was <5% in CoFe_2_O_4_/H_2_O_2_, indicating that the main contribution to oxidation was due to the activation of PAA.

Figure 1c shows that after five cycles of catalytic oxidation, the removal rate of IPM in the catalyst system decreased by 8.7%, indicating that the catalyst has good recycling performance. The cobalt and iron element leaching was investigated in this study. Figure 1d illustrates that the metal leaching increased as the reaction progressed. The leaching concentration of Co was 76.6 μg/L at the optimal parameters, which was significantly lower than the permissible limit of 1 mg/L set by the Chinese National Emission Standard of Pollutants for Copper, Nickel, and Cobalt Industry (GB 25467-2010). The leaching concentration of Fe was slightly less than that of Co (58.4 μg/L), which was consistent with the rule that the B-position element in the spinel structure is more stable than the A-position element [17]. The leaching concentration of Fe was less than the limit of 300 μg/L set by the Chinese Standards of Drinking Water Quality (GB 5749-2022). It suggested that CoFe_2_O_4_/PAA was safe in the water treatment process.

### 3.3. Determination of ROS in CoFe_2_O_4_/PAA

**Figure 2:**
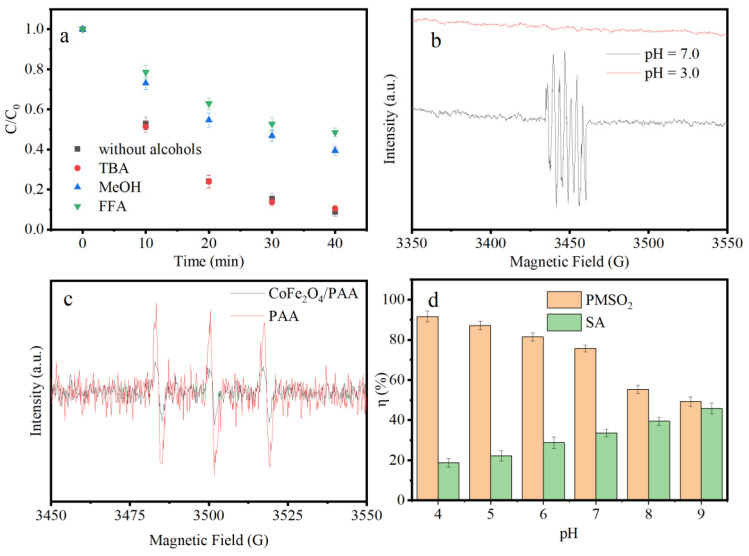
Effect of various quenching agents on IPM degradation of CoFe_2_O_4_/PAA (**a**), in situ ESR spectra of CoFe_2_O_4_/PAA (**b**,**c**), and conversion percentage (η) of PMSO_2_ and SA (**d**). Conditions: pH = 7 in (**a**,**c**,**d**), [PAA] = 200 μM, dosage of CoFe_2_O_4_ = 300 mg/L, [IPM] = 2 μM in (**a**), [TBA] = [MeOH] = [FFA] = 5 mM, [DMPO] = [TEMPO] = 50 mM, [PMSO] = [BA] = 10 μM.

Radical inhibitors were added to investigate the dominant ROS and the contribution of different radicals to the IPM oxidation in the CoFe_2_O_4_/PAA system. Tert-butyl alcohol (TBA) and methanol (MeOH) were employed to test the contribution of OH [24]. As illustrated in Figure 2a, the inhibition of excess TBA (5 mM, 2500 times of IPM) was negligible. Furthermore, 5 mM MeOH did not completely inhibit the degradation of IPM, suggesting that **·**OH was not the main ROS in CoFe_2_O_4_/PAA. Furfuryl alcohol (FFA) was employed to determine the contribution of ^1^O_2_ [25]. The IPM removal decreased by 42.6% in the presence of 5 mM FFA. Similar to MeOH, the degradation of IPM was not completely inhibited in 5 mM FFA, indicating that ^1^O_2_ was not the dominant ROS in CoFe_2_O_4_/PAA.

It has been reported that DMPO is typically oxidized to hydroxylated DMPO or methylated DMPO in the radical oxidation process of UV/PAA [26]. However, the corresponding characteristic peaks of the DMPO-HO**•** and DMPO-CH_3_**•** adducts did not appear in the ESR spectrum (Figure 2b), indicating that the radicals were not the dominant ROS in CoFe_2_O_4_/PAA. In Figure 2b, a characteristic peak of 1:2:1:2:1:2:1 representing 5-dimethyl-1-pyrrolidone-N-oxyl (DMPOX) [27] is observed at pH = 7.0, indicating that the primary ROS can oxidize DMPO to DMPOX under neutral conditions. In the Co(II)/PMS system, the characteristic peak of DMPOX was observed. Similar to the findings of this study, DMPOX formation was highly pH-dependent [27,28], and no ESR signal was observed at pH = 3.0. Co(II)–peroxy is postulated to be generated at a neutral pH and responsible for DMPOX formation [29]. Although the formation mechanism of DMPOX remains unclear, the different ESR signals at pH 3.0 and 7.0 reveal the oxidation characteristics of the CoFe_2_O_4_/PAA system. In addition, previous studies have indicated that the characteristic peak of the DMPO-HO**•** adduct in Co(III) solution at pH 3.0 appeared in the ESR spectrum [28]. The absence of the DMPO-HO**•** adduct at pH 3.0 suggests that a high concentration of Co(III)_aq_ was not generated in CoFe_2_O_4_/PAA.

2,2,6,6-tetramethyl-1-piperidinyloxy (TEMPO) was employed as the ^1^O_2_ trapping agent, and the characteristic peak of TEMP-^1^O_2_ with an intensity ratio of 1:1:1 was observed both with and without CoFe_2_O_4_ [30]. The peak intensity of CoFe_2_O_4_/PAA was considerably lower than that of PAA alone, indicating that the ^1^O_2_ generation in CoFe_2_O_4_/PAA was more inhibited than in PAA alone (Figure 2c). Considering the negligible degradation of IPM, it can be concluded that ^1^O_2_ is not the dominant ROS in CoFe_2_O_4_/PAA.

It has been reported that PAA is similar to PMS and can form a coupling system with Co [31,32]. The Co(II)-PAA complex (Co(II)-OO(O)CCH_3_) has been demonstrated to oxidize organic compounds through the oxygen-atom transfer and single-electron transfer pathways [31]. Sulfoxides can be oxidized to sulfones through the oxygen-atom transfer pathway [33]; therefore, herein, methyl phenyl sulfoxide (PMSO) was selected as the probe for this pathway. The yield of methyl phenyl sulfone (PMSO_2_), denoted as η_PMSO2_, represents the ratio of the generated PMSO_2_ to the consumed PMSO. In CoFe_2_O_4_/PAA, η_PMSO2_ decreased from 91.6% to 49.2% as the pH increased from 4.0 to 9.0 (Figure 2d, details in Figure S4). In contrast, in UV/PAA, η_PMSO2_ was 0 for the same pH range (Figure S5). These results demonstrate that in CoFe_2_O_4_/PAA, the oxygen-atom transfer pathway was the dominant PMSO degradation process under acidic pH conditions. As the pH was increased, the contribution of the oxygen-atom transfer pathway gradually decreased. Notably, the oxygen-atom transfer pathway was absent from the radical oxidation system (UV/PAA).

Furthermore, an η_PMSO2_ value of <1 indicates that some PMSO was oxidized to either hydroxylated MPSO or biphenyl through a single-electron transfer pathway [34]. Therefore, benzoic acid (BA) was oxidized to salicylic acid (SA) as a chemical probe to investigate this pathway, with η_SA_ representing the yield of SA [34,35]. In CoFe_2_O_4_/PAA, η_SA_ increased from 18.7% to 45.8% as the pH was increased from 4.0 to 9.0 (Figure 2d, details in Figure S6), indicating an increased contribution from the single-electron transfer pathway as the pH was increased. This is consistent with the characteristics of the Co(II)-OO(O)CCH_3_ and ≡Cu-OOSO_3_^−^ complexes [31,34], suggesting that the ≡Co(II)-PAA (≡Co(II)-OO(O)CCH_3_) metastable intermediate is the dominant ROS in CoFe_2_O_4_/PAA. However, high-valent metal-oxo species can also convert PMSO into PMSO_2_ through the single-electron transfer pathway [36,37]. Therefore, the generation of high-valent Co and Fe species should be considered in catalytic oxidation.

### 3.4. Electron Transfer Mechanism

**Figure 3:**
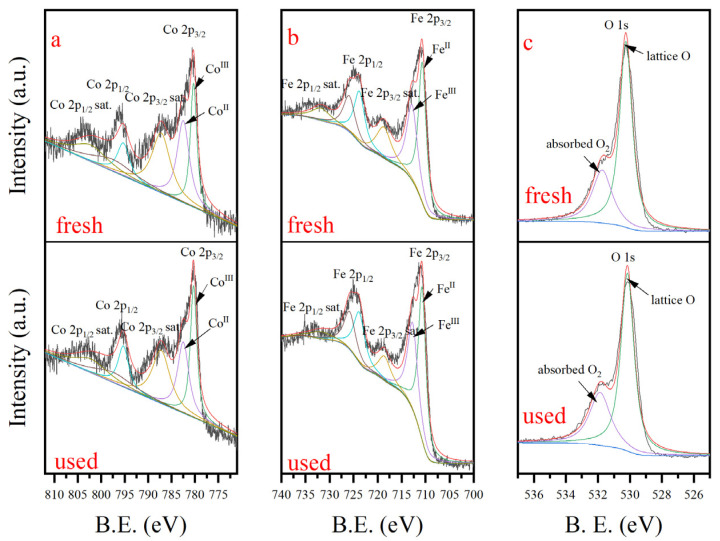
XPS spectra of catalyst before and after reaction (**a**) spectra of Co; (**b**) spectra of Fe; (**c**) spectra of O.

To illustrate the elemental valence changes during catalytic redox, the XPS spectra of the Co, Fe, and O binding energy were analyzed before and after the reaction. The Co 2p3/2 peak exhibited binding energies of 780.3 eV and 782.5 eV, corresponding to Co (III) and Co (II), respectively [38,39]. In the fresh catalyst, Co (III) and Co (II) accounted for 44.26% and 55.74%, respectively. In the used CoFe_2_O_4_, the Co (II) content slightly decreased to 48.29%, while the Co (III) content increased to 51.71% (Figure 3a). The Fe 2p3/2 peak demonstrated binding energies at 710.7 and 712.7 eV, representing Fe (II) and Fe (III), respectively [40]. The fresh CoFe_2_O_4_ had an Fe (II) content of 57.04%, which decreased by only 0.32% to 56.72% after oxidation (Figure 3b), similar to CoFe_2_O_4_/PMS activation [41]. These findings suggest that Co (II) acted as an electron donor in the catalytic oxidation process, while little Fe (II)/Fe (III) redox activity occurred on the catalyst surface. In addition, no evidence of the generation of Co (IV) or high-valent Fe species was observed in the XPS spectrum.

The XPS spectra of O 1s (Figure 3c) exhibited peaks at binding energies of 530.3 and 531.9 eV, representing the content of surface lattice oxygen and adsorbed oxygen, respectively [42]. After the reaction, the lattice oxygen content decreased from 70.9% to 67.8%, while the adsorbed oxygen content increased from 29.1% to 32.2%. This change can be attributed to the interaction between oxygen and cobalt, where part of the lattice oxygen transferred electrons to Co (III), resulting in Co (II) regeneration [43,44].

**Figure 4:**
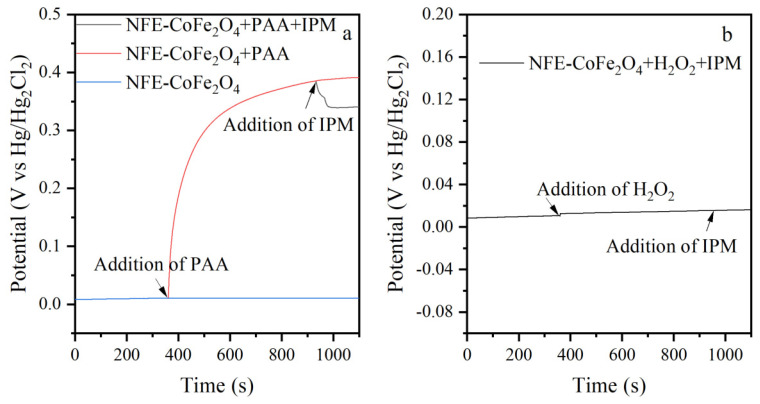
Open-circuit potential curves of the NFE-CoFe_2_O_4_ electrode. (**a**) NFE-CoFe_2_O_4_ electrode with added PAA; and (**b**) NFE-CoFe_2_O_4_ electrode with added H_2_O_2_. Conditions: [PAA] = 200 μM, [H_2_O_2_] = 276 μM, [IPM] = 2 μM, and pH = 7.0.

The open-circuit potential curves were obtained to reveal the electron transfer mechanism between PAA, CoFe_2_O_4_, and IPM. The open-circuit potential of NFE coated with CoFe_2_O_4_ (NFE-CoFe_2_O_4_) was approximately +0.011 V. This potential increased immediately upon PAA addition and stabilized at approximately +0.39 V (Figure 4a), representing the potential of the surface metastable intermediate formed by the interaction between CoFe_2_O_4_ and PAA [21]. In contrast, the potential change of NFE-CoFe_2_O_4_ with the addition of H_2_O_2_ was negligible (Figure 4b), indicating minimal electron transfer in ≡Co (II)-H_2_O_2_ generation [45]. After IPM addition, the potential substantially decreased, suggesting that the surface metastable intermediate disintegrated when reacting with IPM. This analysis highlights the important role of CoFe_2_O_4_ in facilitating the nonradical electron transfer pathway.

Based on the experimental results, the following electron transfer pathway in CoFe_2_O_4_/PAA was proposed. ≡Co (II) served as the primary reactive site, forming the ≡Co (II)-OO(O)CCH_3_ metastable intermediate through an intramolecular electron rearrangement. The metastable intermediate reacted with organic contaminants via the oxygen-atom transfer and single-electron transfer pathways. As the reaction pH was increased, the contribution of the oxygen-atom transfer pathway decreased while that of the single-electron transfer pathway increased. Under neutral conditions, oxygen-atom transfer was the dominant pathway, indicating that radicals and ^1^O_2_ were not the major ROS in CoFe_2_O_4_/PAA.

### 3.5. Estimation of MIAA Formation Potential (MIAAFP)

**Figure 5:**
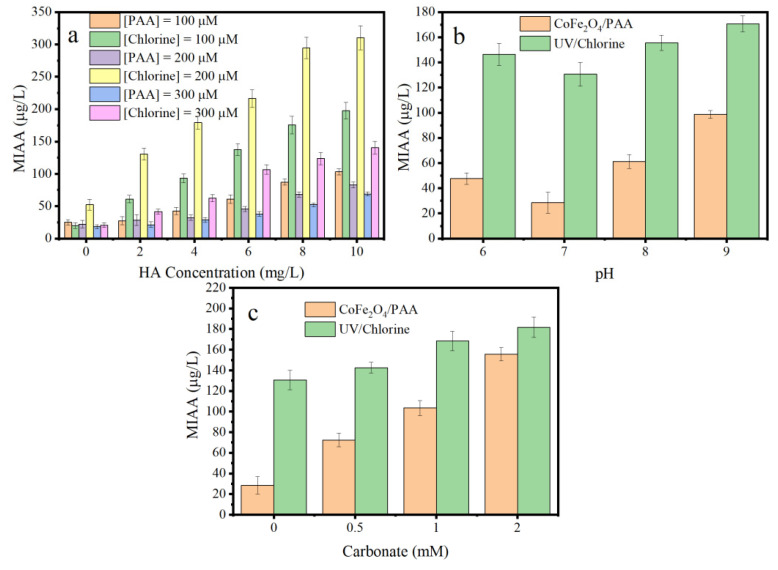
Effect of the oxidant dosage and HA concentration (**a**), pH values (**b**), and carbonate concentration (**c**) for the MIAA formation potential in the CoFe_2_O_4_/PAA and UV/chlorine. Conditions: the dosage of CoFe_2_O_4_ = 300 mg/L, the average fluency rate of UV_254_ = 1.54 mW/cm^2^, and pH = 7.0 in (**a**); [PAA] = [chlorine] = 200 μM, the dosage of CoFe_2_O_4_ = 300 mg/L, and [HA] = 2 mg/L in (**b**); [PAA] = [chlorine] = 200 μM, the dosage of CoFe_2_O_4_ = 300 mg/L, [HA] = 2 mg/L and pH = 7.0 in (**c**); and the reaction time for the CoFe_2_O_4_/PAA and the UV/chlorine was 40 min and 10 min, respectively.

To estimate the control capacity of MIAAFP, CoFe_2_O_4_/PAA was compared with the widely used UV/chlorine in water disinfection treatment, which generates HO• and other radicals (Cl•, Cl_2_•^−^, and ClO•^−^) [46]. HA was selected as the model DOM. As the concentration of HA increased, the MIAAFP increased considerably in both CoFe_2_O_4_/PAA and UV/chlorine. In particular, the MIAAFP in the CoFe_2_O_4_/PAA was considerably lower than that in UV/chlorine at the same HA concentration (0–10 mg/L) and oxidant dosage (100–300 μM, Figure 5a), indicating the superior MIAAFP control capacity of CoFe_2_O_4_/PAA. When the HA concentration was below 4 mg/L, the decrease in the MIAAFP caused by increased oxidant concentration was negligible. However, when the HA concentration was above 4 mg/L, the increase in the MIAAFP was greatly inhibited by the increased PAA dosage. The MIAAFP decreased with an increasing PAA dosage in CoFe_2_O_4_/PAA. In contrast, in UV/chlorine, the MIAAFP followed the trend 300 μM < 100 μM < 200 μM for the chlorine dosage (Figure 5a). Furthermore, the MIAAFP in CoFe_2_O_4_/PAA was lower than that in UV/chlorine at the same oxidant dosage. For example, at an oxidant dosage of 200 μM, the MIAAFP in CoFe_2_O_4_/PAA was 42.7%, 21.8%, 18.0%, 21.0%, 23.1%, and 26.7% of that in UV/chlorine at an HA concentration of 0–10 mg/L.

The MIAAFP followed the trend pH 7.0 < pH 6.0 < pH 8.0 < pH 9.0 in both CoFe_2_O_4_/PAA and UV/chlorine (Figure 5b). Furthermore, the MIAAFP in CoFe_2_O_4_/PAA was considerably lower than that in UV/chlorine. In particular, at pH 7.0, the MIAAFP in CoFe_2_O_4_/PAA was 28.5 μg/L, only 21.8% of that in UV/chlorine, demonstrating the strong MIAAFP control capacity. As illustrated in Figure 5c, carbonate substantially influenced the MIAAFP in CoFe_2_O_4_/PAA. In the presence of 2 mM carbonate, the MIAAFP reached 155.6 μg/L, almost 5.5 times that in the absence of carbonate. Similarly, in UV/chlorine, the MIAAFP increased with the carbonate concentration; however, the increase was not as high as in CoFe_2_O_4_/PAA. Compared to the carbonate-free condition, the MIAAFP increased by 39.1% at a carbonate concentration of 2 mM.

**Figure 6:**
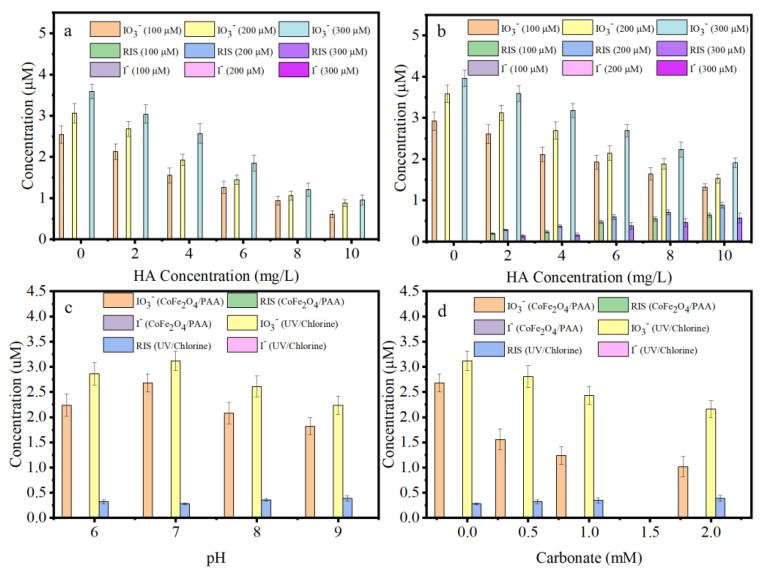
Effect of the oxidant dosage and HA concentration on the inorganic iodine species distribution in the CoFe_2_O_4_/PAA (**a**), and the UV/chlorine (**b**); effect of the pH values (**c**) and carbonate concentration (**d**) on the inorganic iodine species distribution in the CoFe_2_O_4_/PAA and the UV/PAA. Conditions: the dosage of CoFe_2_O_4_ = 300 mg/L, pH = 7.0 in (**a**); the average fluency rate of UV_254_ = 1.54 mW/cm^2^, pH = 7.0 in (**b**); [PAA] = [chlorine] = 200 μM, the dosage of CoFe_2_O_4_ = 300 mg/L, the average fluency rate of UV_254_ = 1.54 mW/cm^2^, and [HA] = 2 mg/L in (**c**,**d**), pH = 7.0 in (**d**); and the reaction time for the CoFe_2_O_4_/PAA and the UV/Chlorine was 40 min and 10 min, respectively.

As illustrated in Figure 6a, the IO_3_^−^ concentration decreased sharply with an increasing HA concentration in CoFe_2_O_4_/PAA due to the inhibition of IPM degradation in the presence of HA (Figure S7a). In contrast, the IO_3_^−^ concentration increased with the PAA dosage due to the enhanced IPM degradation (Figure S7b). In addition, I^−^ from IPM dehalogenation was completely converted into IO_3_^−^ without the formation of RIS, suggesting that the ≡Co (II)-OO(O)CCH_3_ intermediate can oxidize I^−^ to IO_3_^−^ without other inorganic iodine species, enabling MIAAFP control in CoFe_2_O_4_/PAA.

The IO_3_^−^ concentration in UV/chlorine was higher than that in CoFe_2_O_4_/PAA. Furthermore, the RIS concentration increased with an increasing HA concentration (Figure 6b). There was no RIS generation in the absence of HA, suggesting that HO• can convert I^−^ into IO_3_^−^. However, the competition between HO• and HA inhibited the conversion of I^−^ to IO_3_^−^. In addition, the RIS concentration followed the trend of 300 μM < 100 μM < 200 μM for the chlorine dosage, consistent with the trend observed for the MIAAFP, indicating that the MIAAFP is dependent on the RIS concentration. When the oxidant dosage was 200 μM, the total concentration of inorganic iodine species ([IO_3_^−^] + [I^−^] + [RIS]) in UV/chlorine was 3.58, 3.40, 3.06, 2.73, 2.59, and 2.41μM in the presence of 0–10 mg/L HA. These values were 1.17-, 1.27-, 1.58-, 1.88-, 2.44-, and 2.73-fold of those in CoFe_2_O_4_/PAA, respectively. This indicates that dehalogenation was not the key factor in the MIAAFP control. Instead, avoiding RIS generation effectively eliminated the MIAAFP.

The total concentration of inorganic iodine species followed the trend pH 9.0 < pH 8.0 < pH 6.0 < pH 7.0 in both CoFe_2_O_4_/PAA and UV/chlorine (Figure 6c). This was because the inhibition of IPM degradation followed the same rule in the two oxidation processes (Figure S8). The PAA decomposition rate increased with the pH increasing (Figure S9), while H^+^ suppressed the PAA decomposition in CoFe_2_O_4_/PAA, consistent with the observations in Co(II)/PAA [15]. However, this pattern did not follow the trend of IPM degradation and dehalogenation, indicating that the ≡Co (II)-OO(O)CCH_3_ intermediate remained active under neutral conditions.

It has been reported that HO• can oxidize CO_3_^2−^/HCO_3_^−^ to CO_3_•^−^ [27,47], and CO_3_**•**^−^ is a selective oxidant (*E*^0^ = 1.78 V) with a lower redox potential than HO**•** [48]. CO_3_**•**^−^ reacts rapidly with compounds containing electron-rich groups, such as phenol and aniline [49,50]. However, the reaction rate constants between CO_3_**•**^−^ and certain refractory contaminants are low, including atrazine (9.39 × 10^6^ M^−1^ s^−1^), nalidixic acid (7.49 × 10^6^ M^−1^ s^−1^), and caffeine (6.09 × 10^6^ M^−1^ s^−1^) [27]. As a typical refractory ICM, the IPM degradation in UV/chlorine was inhibited in the presence of carbonate because HO**•** was partially transformed into CO_3_**•**^−^ (Figure 6d). In addition, the inhibition of carbonate was stronger in CoFe_2_O_4_/PAA than in UV/chlorine, particularly at high carbonate concentrations. As illustrated in Figure S10, the PAA decomposition was suppressed with the increased carbonate concentration, which may be ascribed to the fact that ≡Co (II) complexed with carbonate, which occupied major reactive sites and blocked the generation of the ≡Co (II)-OO(O)CCH_3_ intermediate. Therefore, the MIAAFP control capacity and dehalogenation in CoFe_2_O_4_/PAA were inhibited (Figure 5c and Figure 6d).

### 3.6. Proposed IPM Degradation Pathway

The 13 IPM transformation products (TPs) detected are presented in Table S4. Of these, only six TPs were generated in CoFe_2_O_4_/PAA, while all the TPs were detected in UV/chlorine (Table S5). In CoFe_2_O_4_/PAA, only one dehalogenation TP (TP-595) was observed. In contrast, four dehalogenation TPs (TP-595, TP-556, TP-625, and TP-609) were detected in UV/chlorine. Notably, no di-dehalogenation TP (TP-556) was formed in CoFe_2_O_4_/PAA. These findings indicated that the traditional AOP (UV/chlorine) exhibited stronger dehalogenation capacity than CoFe_2_O_4_/PAA. This is consistent with the total inorganic iodine species generation pattern, suggesting that IPM dehalogenation was the source of the inorganic iodine species.

**Figure 7:**
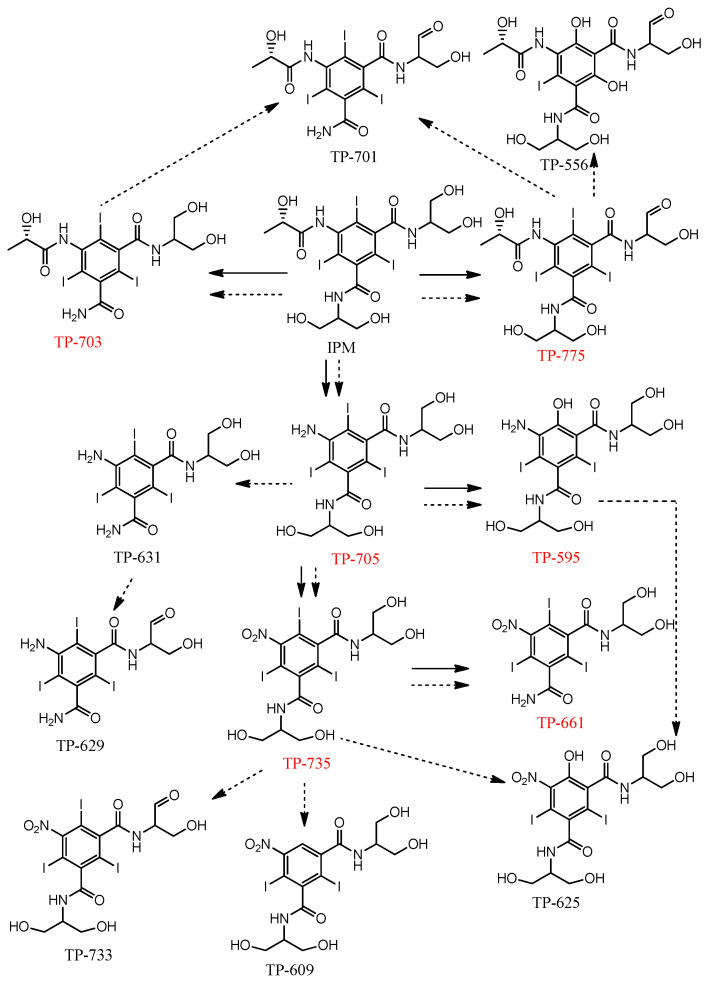
Proposed IPM degradation pathways in the CoFe_2_O_4_/PAA and the UV/chlorine. The red font represents the TP detected in the CoFe_2_O_4_/PAA. The solid and dashed arrows represent the degradation pathways in the CoFe_2_O_4_/PAA and the UV/chlorine, respectively.

As illustrated in Figure 7, IPM underwent three degradation pathways in both oxidation processes: hydrogen abstraction (forming TP-775), dealkylation (forming TP-703), and deacetylation (forming TP-705). These transformation pathways are commonly observed in ICM degradation by AOPs [20,51]. The difference between the two oxidation processes lies in the secondary and tertiary transformation pathways. In CoFe_2_O_4_/PAA, TP-775, and TP-703 did not undergo further degradation. In contrast, these primary TPs were converted into secondary TPs (TP-701 and TP-556) in UV/chlorine. TP-705 was converted into TP-595 and TP-735 in CoFe_2_O_4_/PAA, with TP-595 being the only dehalogenation TP, and not undergoing further degradation. There was only one type of tertiary TP (TP-661), which was the dealkylation product of TP-735.

Compared to CoFe_2_O_4_/PAA, UV/chlorine produced more secondary and tertiary TPs. TP-703 and TP-775 were further degraded into secondary TPs (TP-701 and TP-556) via hydrogen abstraction. Apart from TP-595, TP-705 formed TP-631 through dealkylation. TP-631 and TP-595, as secondary TPs, were converted to TP-629 and TP-625, respectively. In addition to being transformed into TP-661, TP-735 formed TP-733, TP-609, and TP-625 via hydrogen abstraction, dehalogenation, and hydroxylation, respectively. This illustrates the nonselective oxidation of UV/chlorine.

### 3.7. MIAAFP Control in Real Water

**Figure 8:**
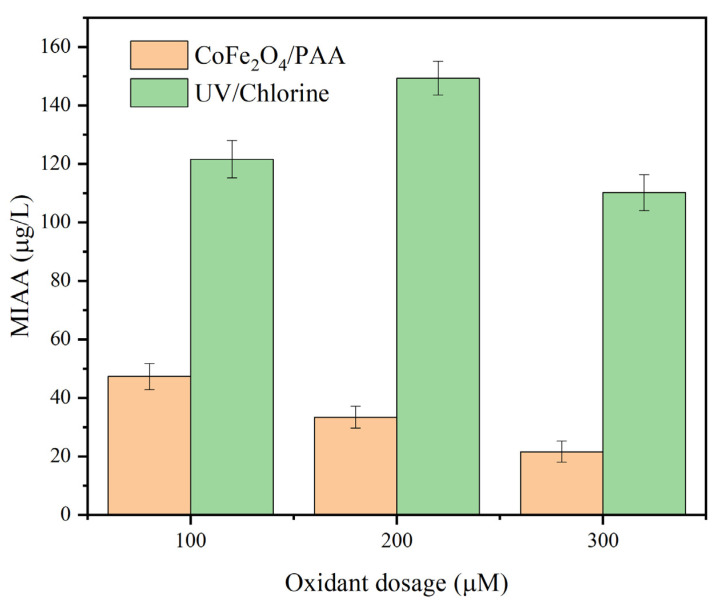
MIAAFP control in real water. Conditions: CoFe_2_O_4_ = 300 mg/L, the average fluency rate of UV_254_ = 1.54 mW/cm^2^, [IPM] = 2 μM, and pH = 6.5.

As illustrated in Figure 8, the MIAAFP decreased as the PAA dosage increased. The MIAAFP was 47.3 μg/L when the PAA dosage was 100 μM. When the PAA dosage was 300 μM, the MIAAFP was 21.6 μg/L. Correspondingly, the MIAAFP rose from 121.6 μg/L to 149.3 μg/L as the chlorine dosage increased from 100 μM to 200 μM. When the chlorine dosage reached 300 μM, the MIAAFP decreased to 110.2 μM. Thus, the MIAAFP control capacity of CoFe_2_O_4_/PAA was significantly higher than that of the UV/chlorine in real water treatment.

## 4. Conclusions

This study demonstrated that IPM was removed efficiently in CoFe_2_O_4_/PAA at a neutral pH. The MIAAFP in CoFe_2_O_4_/PAA was considerably lower than that in UV/chlorine, as the I^−^ from IPM dehalogenation was fully converted into IO_3_^−^ without the formation of RIS in CoFe_2_O_4_/PAA. The MIAAFP control capacity of CoFe_2_O_4_/PAA was significantly higher than that of the UV/chlorine in real water treatment. Thus, CoFe_2_O_4_/PAA is a promising treatment for removing ICMs and controlling the formation of I-DBPs.

## Data Availability

Data is contained within the article or Supplementary Material.

## Supplementary Materials

The following supporting information can be downloaded at https://www.mdpi.com/article/10.3390/nano15120897/s1, Figure S1: The size distribution of the CoFe_2_O_4_ particle; Figure S2: XRD spectrum of CoFe_2_O_4_ (a); N_2_ adsorption-desorption isotherms and pore size distribution of CoFe_2_O_4_ (b); Figure S3: The IPM degradation in the CoFe_2_O_4_/H_2_O_2_; Figure S4: The η_PMSO2_ variations in the CoFe_2_O_4_/PAA; Figure S5: The η_PMSO2_ variations in the UV/PAA. Figure S6: The η_SA_ variations in the CoFe_2_O_4_/PAA; Figure S7: The IPM degradation variations with the HA concentration (a) and PAA dosage (b); Figure S8: The IPM degradation variations with pH in the two oxidation processes; Figure S9: The PAA decomposition with pH in the CoFe_2_O_4_/PAA; Figure S10: The PAA decomposition with the carbonate concentration in the CoFe_2_O_4_/PAA; Figure S11: The accurate mass profiles of IPM and the transformation products; Table S1: Chemical reagents and materials; Table S2: Water quality parameters of the real water; Table S3: HPLC conditions for target compounds analysis; Table S4: Transformation products of IPM oxidation; Table S5: The distribution of transformation products of IPM oxidation in two oxidation processes; Text S1: The RIS capture experiment procedure; Text S2: Analytical methods for transformation products; Text S3: Analytical methods for MIAA.
