# Supplementary material for: Insights into CoFe2O4/Peracetic Acid Catalytic Oxidation Process for Iopamidol Degradation: Performance, Mechanisms, and I-DBP Formation Control
Source: Nanomaterials (Basel). 2025 Jun 10;15(12):897. doi: 10.3390/nano15120897 (PMC12195921; doi:10.3390/nano15120897)
Supplement: Supplementary file 1 [file nanomaterials-15-00897-s001.zip › nanomaterials-3685723-supplementary.pdf]

Supporting Information

for

Insights into $CoFe_2O_4$/ peracetic acid catalytic oxidation process for iopamidol degradation: performance, mechanisms, and I-DBP formation control

Haiwei Wu [1], Jiaming Zhang [2]*, Fangbo Zhao [1]**, Wei Fan [2], Song Yang [3], and Jun Ma [4]

[1] College of Material Science and Chemical Engineering, Harbin Engineering University, Harbin 150001, China

[2] School of Environment, Northeast Normal University, 2555 Jingyue Street, Changchun 130117, China

[3] Resources and Environmental Innovation Institute, Shandong Jianzhu University, Jinan 250101, China

[4] School of Environment, Harbin Institute of Technology, Harbin 150001, China

*Corresponding Authors:

*Jiaming Zhang; E-mail: zhangjm@nenu.edu.cn

**Fangbo Zhao; E-mail: zhaofangbo@hrbeu.edu.cn

Table S1-Chemical reagents and materials

Reagent	Standard	Producer
H_2O	GR	Millipore Co., Ltd. (Shanghai, China)
acetic acid(CH_3COOH)	AR	Sinopharm Chemical Reagent (Tianjin, China)
hydrogen peroxide(H_2O_2)	30%	Hengxing Chemical Reagent (Tianjin, China)
sulfuric acid(H_2SO_4)	98%	Kermel Chemical Reagent (Shanghai, China)
$(NH_4)_6Mo_7O_{24}\cdot 4H_2O$	AR	Sinopharm Chemical Reagent (Tianjin, China)
phosphoric acid(H_3PO_4)	85%	Macklin Biochemical (Shanghai, China)
potassium iodide(KI)	GR	Kemiou Chemical Reagent(Shanghai, China)
potassium iodate(KIO_3)	GR	Kemiou Chemical Reagent (Shanghai, China)
iopamidol(IPM)	98%	Aladdin Biological Technology (Shanghai, China)
disodium hydrogen phosphate trihydrate (Na_2HPO_4)	AR	Kemiou Chemical Reagent (Shanghai, China)
sodium dihydrogen ($NaH_2PO_4\cdot 2H_2O$)	GR	Kemiou Chemical Reagent (Shanghai, China)
cobalt ferrite ($CoFe_2O_4$)	99%	Macklin Biochemical (Shanghai, China)
sodium hydroxide(NaOH)	GR	Kemiou Chemical Reagent (Shanghai, China)
$Na_2S_2O_3\cdot 5H_2O$	GR	Kemiou Chemical Reagent (Shanghai, China)
humic acid(HA)	≥ 90%	Aladdin Biological Technology (Tianjin, China)
monoiodoacetic acid (MIAA)	AR	Aladdin Biological Technology (Tianjin, China)
sodium hypochlorite(NaClO)	AR	Kemiou Chemical Reagent (Shanghai, China)
methyl tert-butyl ether(MTBE)	GC	Macklin Biochemical (Shanghai, China)
methanol(MeOH)	GC	Macklin Biochemical (Shanghai, China)

phenol	GR	Kemiou Chemical Reagent (Shanghai, China)
2-iodophenol	98%	Aladdin Biological Technology (Shanghai, China)
4-iodophenol	98%	Aladdin Biological Technology (Shanghai, China)
tert-butyl alcohol (TBA)	98%	Aladdin Biological Technology (Shanghai, China)
humic acid (HA)	AR	Aladdin Biological Technology (Shanghai, China)
5,5-dimethyl-1-pyrroline N-oxide (DMPO)	GR	Aladdin Biological Technology (Shanghai, China)
sodium bicarbonate($NaHCO_3$)	AR	Sinopharm Chemical Reagent (Shanghai, China)
furfuryl alcohol(FFA)	97%	Macklin Biochemical (Shanghai, China)
salicylic acid(SA)	AR	Sinopharm Chemical Reagent (Shanghai, China)
benzoic acid(BA)	AR	Sinopharm Chemical Reagent (Shanghai, China)
methyl phenyl sulfoxide (PMSO)	AR	Aladdin Biological Technology (Shanghai, China)
methyl phenyl sulfone ($PMSO_2$)	AR	Aladdin Biological Technology (Shanghai, China)

Table S2- Water quality parameters of the real water

surface water
$[HCO_3^-] + [H_2CO_3] = 0.18$ mM
$[Cl^-] = 0.23$ mM
$[SO_4^{2-}] = 0.18$ mM
$[HPO_4^{2-}] + [H_2PO_4^-] = 0.02$ mM
TOC = 2.35 mg C/L
$UV_{254nm} = 0.08$
pH = 6.5

Table S3- HPLC conditions for target compounds analysis

Compound	Eluent A	Eluent B	A: B(v/v)	wavelength（nm）
IPM	methanol	water	10:90	243
I^-	acetonitrile	0.09M KH_2PO_4	2:98	226
IO_3^-	acetonitrile	0.1%H_3PO_4	35:65	209
2-iodophenol	methanol	1‰ acetic acid	60:40	280
4-iodophenol	methanol	1‰ acetic acid	60:40	231
PMSO	acetonitrile	water	20:80	230
$PMSO_2$	acetonitrile	water	20:80	215
BA	methanol	water	20:80	227
SA	methanol	water	30:70	305

Table S4- Transformation products of IPM oxidation

Compounds	Retention time (min)	Detected mass (m/z)	Calculated mass (m/z)	Error (ppm)	molecular formula
IPM	4.35	777.861	777.854	8.9	$C_{17}H_{22}I_3N_3O_8$
TP-703	4.67	703.825	703.817	11.4	$C_{14}H_{16}I_3N_3O_6$
TP-701	6.01	701.793	701.802	-12.8	$C_{14}H_{14}I_3N_3O_6$
TP-556	5.83	556.041	556.035	10.8	$C_{17}H_{22}IN_3O_{10}$
TP-775	3.82	775.840	775.838	2.6	$C_{17}H_{20}I_3N_3O_8$
TP-705	5.61	705.840	705.833	9.9	$C_{14}H_{18}I_3N_3O_6$
TP-595	6.96	595.940	595.931	15.1	$C_{14}H_{19}I_2N_3O_7$
TP-631	5.92	631.802	631.796	9.5	$C_{11}H_{12}I_3N_3O_4$
TP-629	5.71	629.790	629.781	14.3	$C_{11}H_{10}I_3N_3O_4$
TP-735	7.19	735.815	735.807	10.9	$C_{14}H_{16}I_3N_3O_8$
TP-661	7.70	661.779	661.770	13.6	$C_{11}H_{10}I_3N_3O_6$
TP-733	7.05	733.783	733.792	-12.3	$C_{14}H_{14}I_3N_3O_8$
TP-625	5.79	625.909	625.905	6.4	$C_{14}H_{17}I_2N_3O_9$
TP-609	5.61	609.917	609.910	11.5	$C_{14}H_{17}I_2N_3O_8$

Table S5-The distribution of transformation products of IPM oxidation in two oxidation processes

No.	Compounds	$CoFe_2O_4$/PAA	UV/Chlorine
1	TP-703	√	√
2	TP-701	-	√
3	TP-556	-	√
4	TP-775	√	√
5	TP-705	√	√
6	TP-595	√	√
7	TP-631	-	√
8	TP-629	-	√
9	TP-735	√	√
10	TP-661	√	√
11	TP-733	-	√
12	TP-625	-	√
13	TP-609	-	√

Text S1- The RIS capture experiment procedure

Because HOI and I_2 can quickly react with the capture reagent phenol to form 2-iodophenol and 4-iodophenol, the concentrations of HOI and I_2 cannot be quantified separately. The oxidation sample without quenching was filtered out of the catalyst by a 0.45 μm fiberglass membrane. The filtrate (0.5 mL) was collected from an HPLC sample bottle with 0.5 mL of phenol solution (20 μM), and the capture reaction was finished in 1 min. The detection conditions of 2-iodophenol and 4-iodophenol are provided in Table S2.

Text S2-Analytical methods for transformation products

The oxidation products of IPM were identified by liquid chromatography-mass spectrometry (AB SCIEX, TripleTOF 5600) equipped with Waters UPLC H-Class system. The mobile phase consisting of pure water (phase A) and methanol (phase B) was eluted via gradient solvent at a 0.4 mL/min flow rate. The volume ratio was initially maintained at 95%: 5% (A: B) for 1 min, then increased linearly to 5%: 95% (A: B) in 12 min and then kept for 4 min. Afterward, the volume ratio reverted to 95%: 5% for 0.1 min and re-equilibrated for 6 min.

Mass spectra (m/z 100–2000 Da) were measured on positive mode electrospray ionization (ESI+) for MS and MS/MS analysis IPM and its transformation products. The operating conditions were: ion spray voltage floating, +5500 V; capillary temperature, 550 ℃; probe heater temperature, 550 ℃; sheath gas, 40 arb; aux gas, 15 arb; S-lens, 50.

Text S3- Analytical methods for MIAA

The method for quantifying MIAA was developed by modifying the USEPA Method 552.2. Samples were extracted by liquid/liquid extraction with MTBE with acidic methanol derivation, and a gas chromatograph (GC, Agilent 8890) equipped with an ECD detector and a gas chromatographic column (Agilent Poroshell 120 EC-18) was employed to analyze the extracts. The injector and detector temperatures were 210 ℃ and 290 ℃, respectively, and the flow rate of the nitrogen carrier gas was 41.9 cm/s with a pressure of 127.7 kPa. The column temperature program for MIAA was as follows: initially hold at 50 ℃ for 1 min, then increased to 150 ℃ at 5 ℃/min and maintained for 4 min, finally ramp to 220 ℃ at 10 ℃/min and maintained for 8 min.

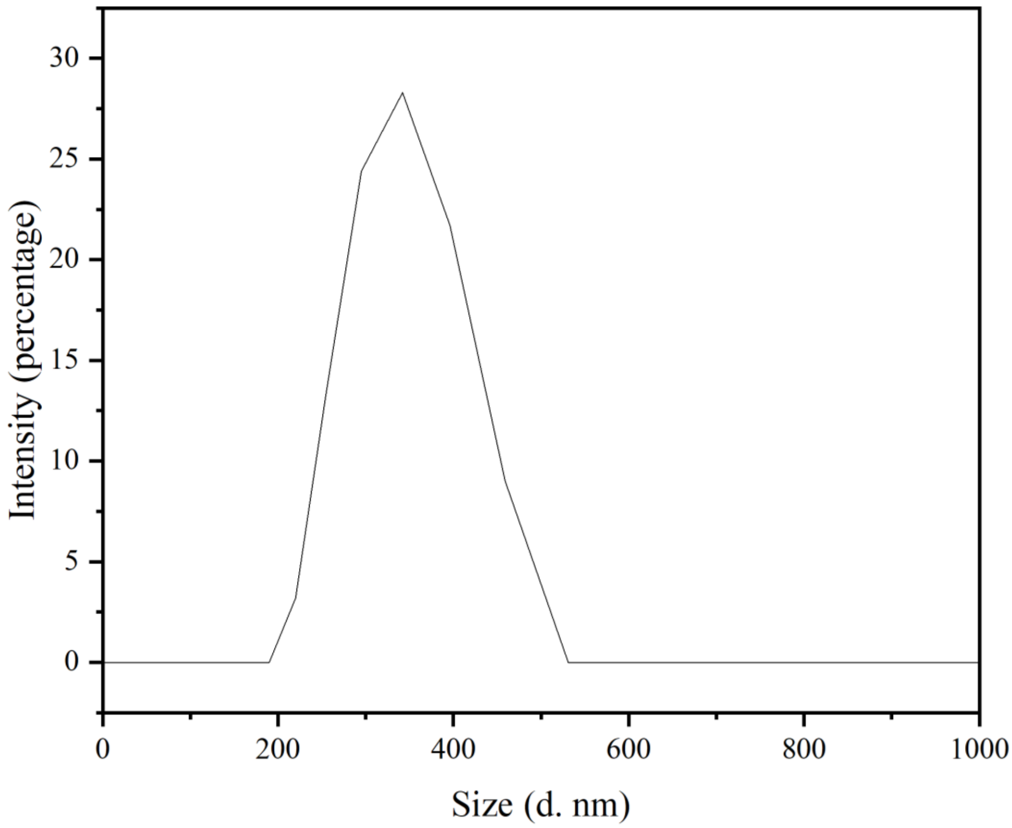

Figure S1-The size distribution of the $CoFe_2O_4$ particle

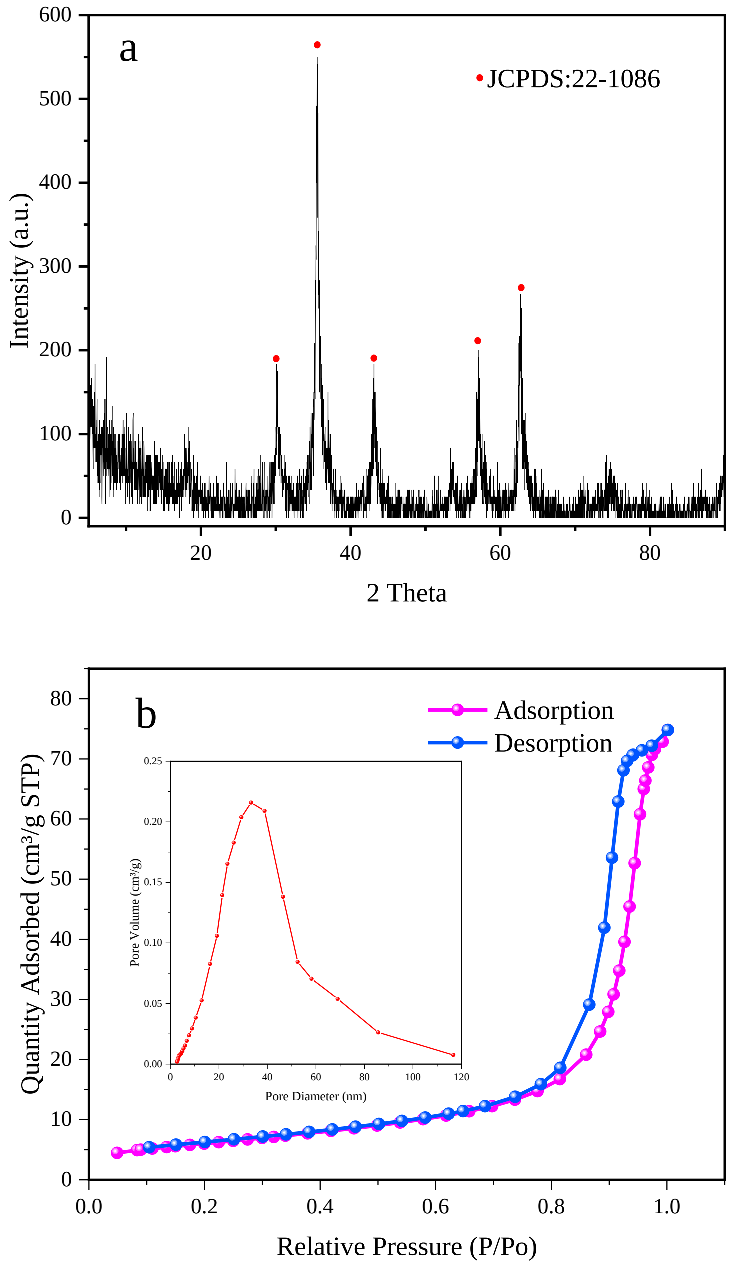

Figure S2- XRD spectrum of $CoFe_2O_4$ (a); N_2 adsorption-desorption isotherms and pore size distribution of $CoFe_2O_4$ (b).

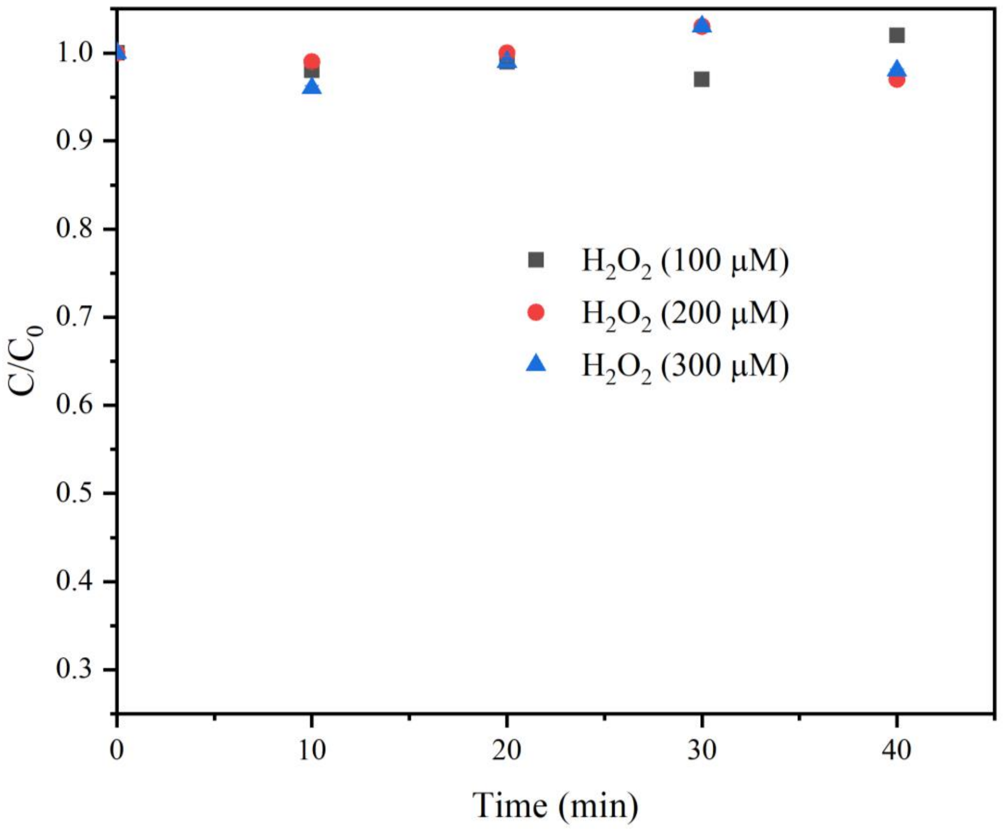

Figure S3-The IPM degradation in the $CoFe_2O_4/H_2O_2$. Conditions: dosage of $CoFe_2O_4$ =300 mg/L, [IPM] = 2 μM, and pH = 7.0.

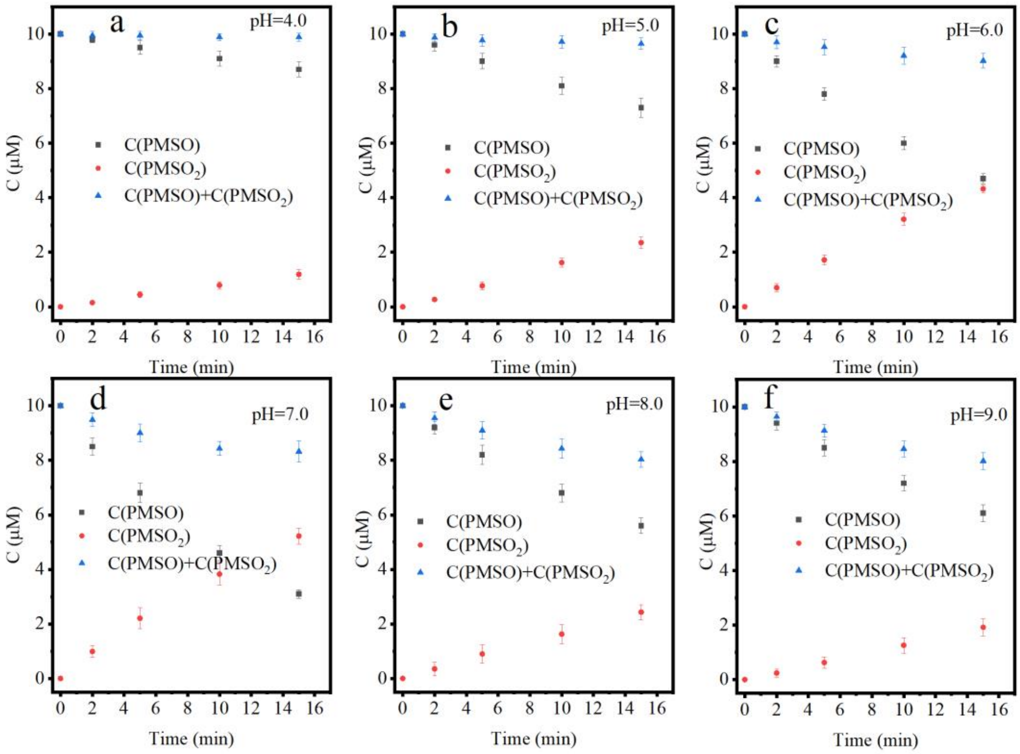

Figure S4-The η_{PMSO2} variations in the $CoFe_2O_4$/PAA. Conditions: [PAA]=200 μM, dosage of $CoFe_2O_4$ =300 mg/L, and [PMSO] = 10 μM.

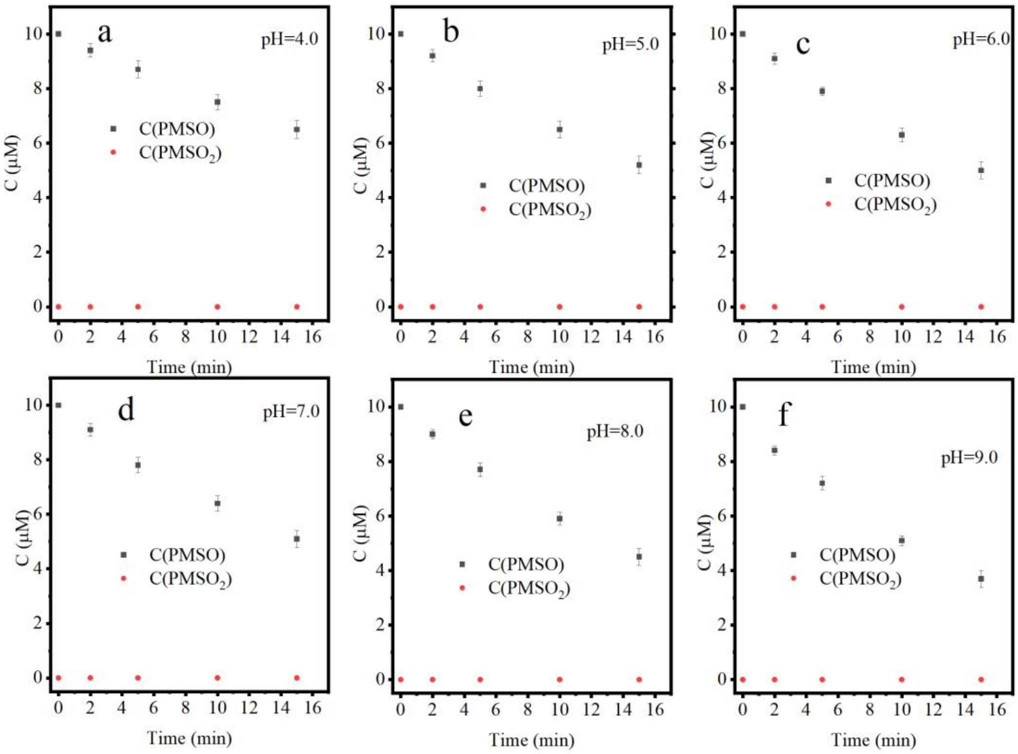

Figure S5-The η_{PMSO2} variations in the UV/PAA. Conditions: [PAA]=200 μM, average fluency rate = 1.54 mW/cm^2, and [PMSO] = 10 μM.

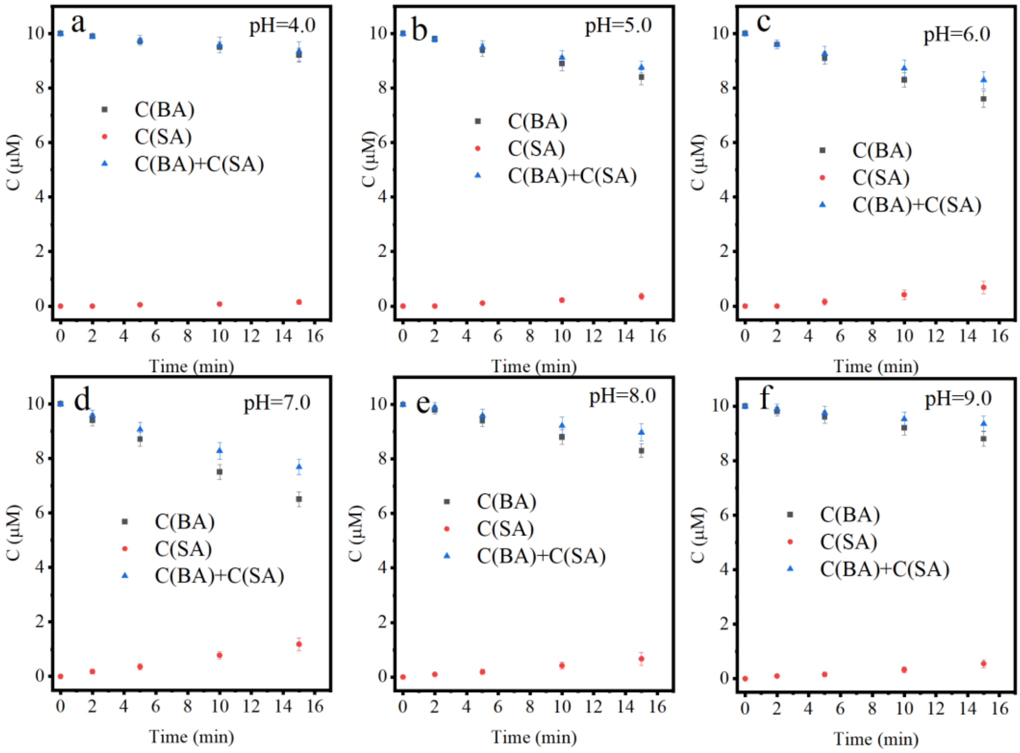

Figure S6- The η_{SA} variations in the $CoFe_2O_4$/PAA. Conditions: [PAA]=200 μM, dosage of $CoFe_2O_4$ =300 mg/L, and [BA] = 10 μM.

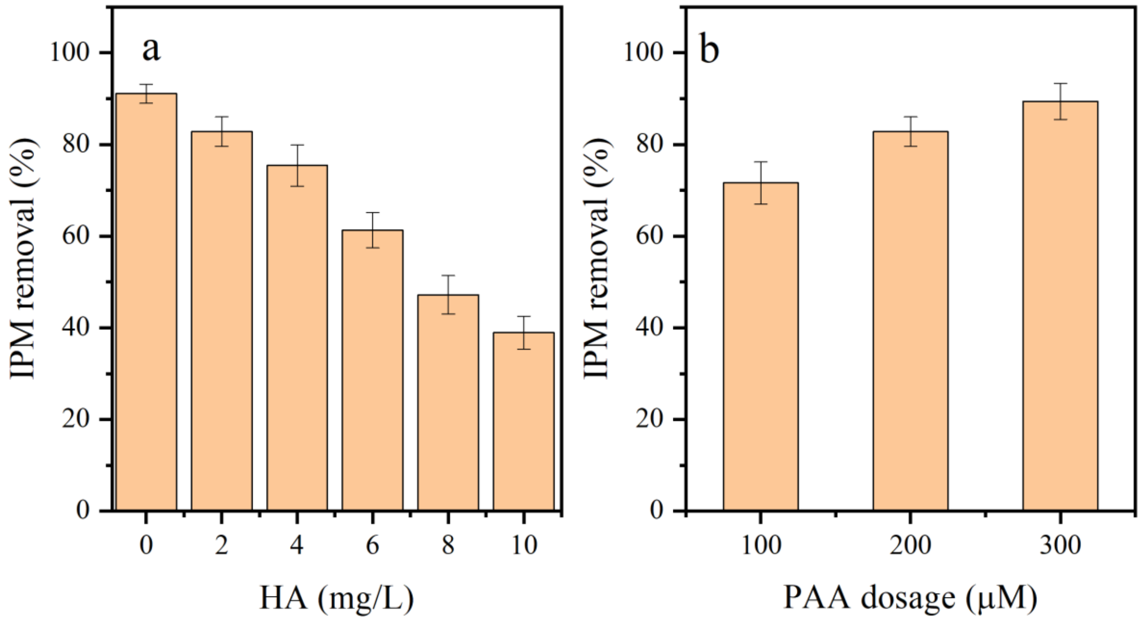

Figure S7-The IPM degradation variations with the HA concentration (a) and PAA dosage (b). Conditions: [PAA]=200 μM, dosage of $CoFe_2O_4$ =300 mg/L, [IPM] = 2 μM, pH= 7.0, and reaction time = 40 min in (a); dosage of $CoFe_2O_4$ =300 mg/L, [IPM] = 2 μM, [HA] = 2 mg/L, and reaction time = 40 min in (b).

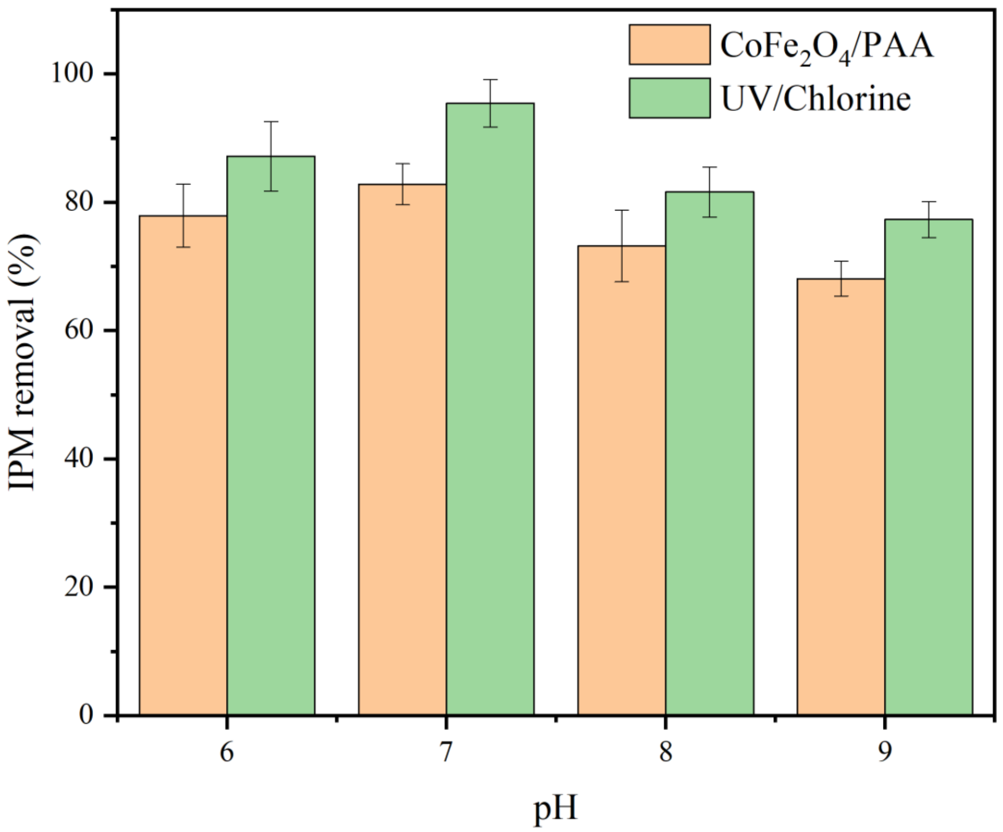

Figure S8- The IPM degradation variations with pH in the two oxidation processes. Conditions: dosage of $CoFe_2O_4$ =300 mg/L, the average fluency rate of UV_{254} = 1.54 mW/cm^2, [HA] = 2 mg/L, and [PAA] = [chlorine] = 200 μM.

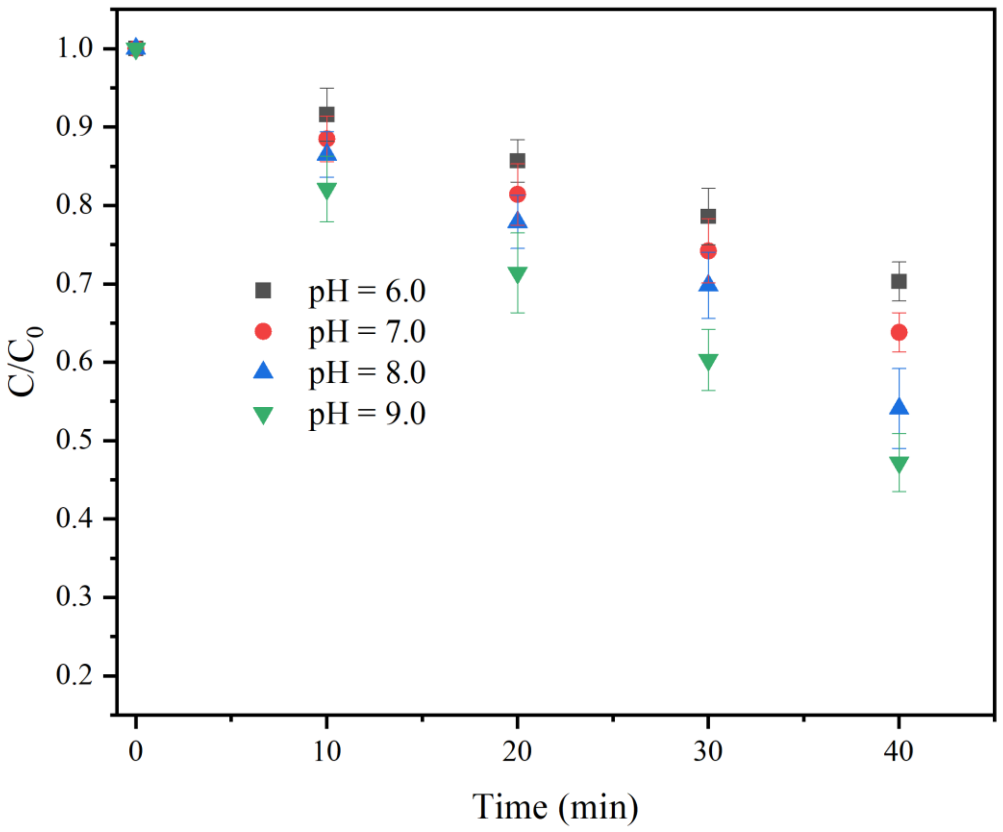

Figure S9-The PAA decomposition with pH in the $CoFe_2O_4$/PAA. Conditions: dosage of $CoFe_2O_4$ =300 mg/L, [PAA] = 200 μM, and [HA] = 2 mg/L.

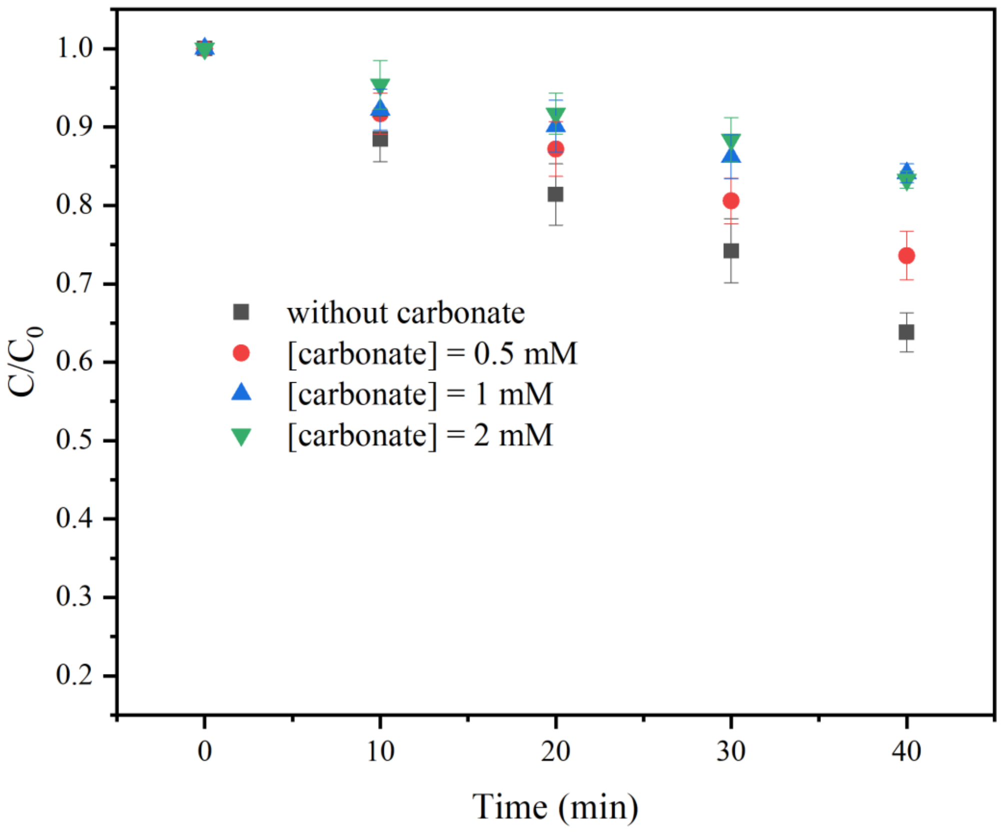

Figure S10- The PAA decomposition with the carbonate concentration in the $CoFe_2O_4$/PAA. Conditions: dosage of $CoFe_2O_4$ =300 mg/L, [PAA] = 200 μM, pH = 7.0, and [HA] = 2 mg/L.

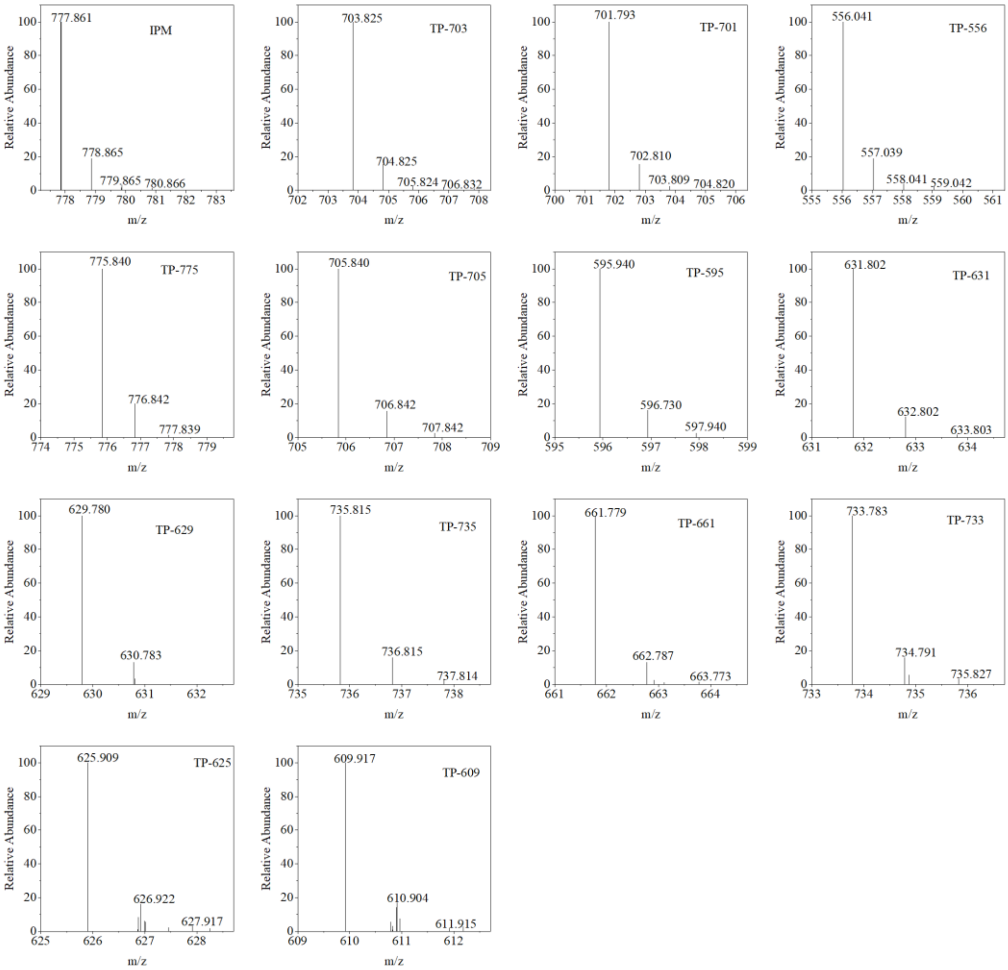

Figure S11- The accurate mass profiles of IPM and the transformation products
